# Monitoring of Sodium Valproate Annual Risk Assessments Within Psychiatric Services

**DOI:** 10.1192/bjo.2024.546

**Published:** 2024-08-01

**Authors:** Melissa Bremner

**Affiliations:** Woodland View Hospital, Irvine, United Kingdom

## Abstract

**Aims:**

To determine the number of patients within a service on sodium valproate for a psychiatric condition who have updated Annual Risk Acknowledgement forms in place.

**Methods:**

It was firstly identified that within the NICE guidelines, it is recommended that all patients who are on sodium valproate should have an annual signed risk acknowledgement form in place. Following this, a list of patients was compiled who were currently prescribed this with the local area. Each patient was then checked to see if the valproate was prescribed by psychiatry or by neurology. This was then further divided into general adult and learning disability patients.

From this, a list of patients under the care of general adult psychiatry was compiled. The notes for these patients were obtained.

Data collection was then carried out. Each set of notes was reviewed by two individuals for the following:

1. To identify if an annual risk assessment form was carried out.
2. To check if this was within expiry date.
3. To identify patient diagnosis.
4. To identify the dose of sodium valproate.
5. To confirm if these patients were females of childbearing age.

**Results:**

From the initial audit cycle, it was identified that 28 female patients who fell within the inclusion criteria were on valproate, and of these, 6 had forms in place. Of the 6 with forms in place, 50% had expired so needed to be replaced. 17 had no form in place, and for 4 patients it could not be certain if forms were present or not due to unavailability of records. Only 3 patients therefore had the correct form in place which were within expiry date. If we discount those with no data available, only 12% of patients had the correct annual risk acknowledgement form present and within expiry date.

Following the initial audit, two interventions were carried out:

1. The data from the above audit was presented at a consultant meeting, highlighting the importance of ensuring these forms are kept up to date.
2. It was decided that pharmacy would take a leading role in ensuring the annual risk assessment forms are updated.

Following 6 months, this patient cohort was re-audited, with further results obtained.

These results showed a reduction in patients prescribed sodium valproate from 28 to 19. 37% of all patients prescribed sodium valproate had forms, but of these, only 30% were up to date. Therefore, only 11% of patients had correct annual risk acknowledgement forms in place which were up to date.

These results showed an improvement in those who had at one time had a form in place, but roughly similar compliance with availability of up to date forms.

**Conclusion:**

Overall it appears that there is a real lack of consistency in ensuring the annual risk assessment forms are in place. A very low percentage of patients have the correct form in place within expiry date, despite interventions which have taken place as part of this audit. Further efforts should therefore be made by teams to ensure that these forms are in place and up to date for all women of childbearing age on valproate.

On a positive note, it may be possible to surmise that the reduction in number of patients on sodium valproate may be linked to raised awareness of the risks to women of childbearing age.

Additionally, a significant number of patients had been sent forms in the post, but had not returned them. Some of these same patients had, in the time that they should have had updated risk forms put in place, been admitted to an inpatient psychiatric hospital. It is therefore important to consider in future if these forms should be updated whilst the patients are admitted to hospital, to increase the number of patients with these forms up to date and therefore improve safety for this cohort of patients. Given that many of these patients have a diagnosis of Bipolar Affective Disorder, they may find it more challenging to return the forms whilst in the community, particularly if unwell; the ICD–11 criteria does note that patients with this condition may suffer from “distractibility, impulsive behaviour and rapid changes in mood state.” Further research could therefore be carried out to determine if utilising inpatient admissions to discuss risks of their medications would be a beneficial way to improve compliance with Annual Risk Assessment Forms.

Lastly, we know that there has been discussion around implementation of such monitoring for men as well as for women. Should this be implemented within the NICE guidelines, further audits should be carried out to determine our compliance with this.

